# Novel Variant of New Delhi Metallo-Beta-Lactamase (*bla*_NDM-60_) Discovered in a Clinical Strain of *Escherichia coli* from the United Arab Emirates: An Emerging Challenge in Antimicrobial Resistance

**DOI:** 10.3390/antibiotics13121158

**Published:** 2024-12-02

**Authors:** Farah Al-Marzooq, Akela Ghazawi, Mushal Allam, Timothy Collyns, Aqeel Saleem

**Affiliations:** 1 Department of Medical Microbiology and Immunology, College of Medicine and Health Sciences, United Arab Emirates University, Al Ain P.O. Box 15551, United Arab Emirates; 2 Department of Genetics and Genomics, College of Medicine and Health Sciences, United Arab Emirates University, Al Ain P.O. Box 15551, United Arab Emirates; 3 Tawam Hospital, Al Ain P.O. Box 5674, United Arab Emirates

**Keywords:** *Escherichia coli*, whole-genome sequencing, New Delhi metallo-β-lactamase, NDM-60, carbapenem resistance, United Arab Emirates

## Abstract

**Background/Objectives:** Carbapenem resistance poses a significant health threat. This study reports the first detection and characterization of a novel variant of New Delhi metallo-β-lactamase (*bla*_NDM-60_) in *Escherichia coli* from the United Arab Emirates (UAE), including its genetic context and relationship to global strains. **Methods:** NDM-60-producing *E. coli* was isolated from a rectal swab during routine screening. Characterization involved whole-genome sequencing, antimicrobial susceptibility testing, and comparative genomic analysis with 66 known NDM variants. Core genome analysis was performed against 42 global *E. coli* strains, including the single other reported NDM-60-positive isolate. **Results:** The strain demonstrated extensive drug resistance, including resistance to novel β-lactam/β-lactamase inhibitor combinations, notably taniborbactam. NDM-60 differs from the closely related NDM-5 by a single amino acid substitution (Asp202Asn) and two amino acid substitutions (Val88Leu and Met154Leu) compared to NDM-1. NDM-60 is located on a nonconjugative IncX3 plasmid. The strain belongs to sequence type 940 (ST940). Phylogenetic analysis revealed high diversity among the global ST940 strains, which carry a plethora of resistance genes and originated from humans, animals, and the environment from diverse geographic locations. **Conclusions:** NDM-60 emergence in the UAE represents a significant evolution in carbapenemase diversity. Its presence on a nonconjugative plasmid may limit spread; however, its extensive resistance profile is concerning. Further studies are needed to determine the prevalence, dissemination, and clinical impact of NDM-60. NDM evolution underscores the ongoing challenge in managing antimicrobial resistance and the critical importance of vigilant molecular surveillance. It also highlights the pressing demand to discover new antibiotics to fight resistant bacteria.

## 1. Introduction

Antimicrobial resistance (AMR) represents one of the most urgent global health crises, threatening millions of lives worldwide. Over the years, the effectiveness of antibiotics has significantly declined due to the emergence of highly resistant bacterial strains. If this trend continues, it is likely that all current antibiotics will eventually become ineffective against multidrug-resistant (MDR) bacteria [1,2]. Beta-lactam antibiotics represent vital and highly prescribed drugs in different parts of the world [3]. Among the beta-lactam antibiotics, carbapenems exhibit the broadest spectrum of activity and the highest potency against MDR bacteria, representing a valuable tool in the antimicrobial armamentarium [4]. Carbapenems act by targeting penicillin-binding proteins (PBPs), thus impeding bacterial cell wall synthesis [5]. These drugs have often been considered as last-resort treatments for MDR infections; however, they are progressively losing their effectiveness. Unfortunately, Gram-negative pathogens produce different classes of beta-lactamases, including carbapenemases, with potent hydrolytic activity against carbapenems [6]. Nowadays, carbapenem-resistant *Enterobacterales* (CREs) have global distribution, posing a serious public health threat [5].

Carbapenem-inactivating carbapenemases are predominantly divided into Class A, B, and D according to the Ambler classification [7]. Classes A and D include serine β-lactamases, and B includes to metallo-β-lactamases (MBLs). MBLs are considered the most potent enzymes, hydrolyzing a wide array of beta-lactam antibiotics. This class relies on metal-mediated hydrolysis, typically through zinc ions, which are needed to catalyze the degradation of β-lactam antibiotics [8,9]. The most clinically relevant carbapenemases include Verona integron-encoded MBL (VIM), imipenemase (IMP) and New Delhi MBL (NDM) [10]. NDM is a typical member of class B1 of MBLs, capable of hydrolyzing all β-lactams, except monobactams [11]. NDM was first identified in 2008 in a *Klebsiella pneumoniae* isolate. Since then, more bacterial isolates producing NDM have been discovered globally [12], posing a significant challenge for treatment, infection control, and public health [13].

The rate of infections caused by NDM-carrying CREs has been significantly increasing. To date, up to 67 variants of NDM have been recognized according to the latest updates provided by the National Center for Biotechnology Information [14]. NDM-1 and NDM-5 are the most frequently detected variants in *Enterobacterales* [5]. Both are commonly encoded on IncX3 plasmids [15]. However, NDM-5 is more prevalent than NDM-1 in *Escherichia coli* [16,17]. Data on other variants of NDM are lacking, as a few strains carrying new alleles have been reported globally, with a paucity of comprehensive genomic analyses of these novel alleles.

In this study, we aimed to characterize the new NDM-60 variant discovered in a single *E. coli* strain from the United Arab Emirates (UAE). We aimed to explore the genetic context of *bla*_NDM-60_ compared to other closely related variants detected by phylogenetic analysis. Additionally, our goal was to elucidate the genetic relatedness of our strain to other strains from the same clone (sequence type) reported in other parts of the world.

## 2. Results

*E. coli* (EC961) was detected on a routine rectal screening for CREs during hospitalization of a patient admitted to Tawam Hospital, Al Ain, UAE, in November 2023. The strain was isolated from a swab taken from a 40-year-old man with no prior medical history who presented to the emergency department with acute-onset bilateral lower limb weakness, sparing sensation, and demonstrating hyporeflexia. Initial brain imaging was unremarkable, and Guillain–Barré syndrome was suspected clinically. He started to have upper limb weakness as well, was shifted to ICU for close monitoring of respiratory functions, and required intubation for respiratory support. Later, he developed respiratory failure, multiorgan dysfunction, and brain edema. Despite aggressive medical management including antimicrobials, immunomodulation, and supportive care, the patient’s condition deteriorated, leading to death.

Whole-genome sequencing (WGS) was employed to obtain a comprehensive overview of the genomic contents of the strain. NDM-60 was identified using NCBI tools, and the strain was tested for antibiotic susceptibility.

### 2.1. Antibiotic Susceptibility

As shown in Table 1, EC961 was highly resistant to multiple antibiotics, except aminoglycosides, and last-resort drugs such as tigecycline, colistin, and cefiderocol. For the latter, the MIC was at the upper border of susceptible range (MIC: 4 µg/mL). The strain was also resistant to some novel beta-lactam/beta lactamase inhibitor combinations such as cefepime/taniborbactam and meropenem/nacubactam (MIC: ≥16 µg/mL) but was susceptible to other combinations such as cefepime/zidebactam and meropenem/xeruborbactam (MIC: 0.5–1 µg/mL). The susceptibility profile was different from the control strain expressing NDM-1, which seems to be more susceptible to the novel beta-lactam/beta lactamase inhibitor combinations and novel cephalosporins such as cefiderocol.

### 2.2. Phylogenetic Relationship of NDM-60 to Other NDM Alleles

**Figure 1:**
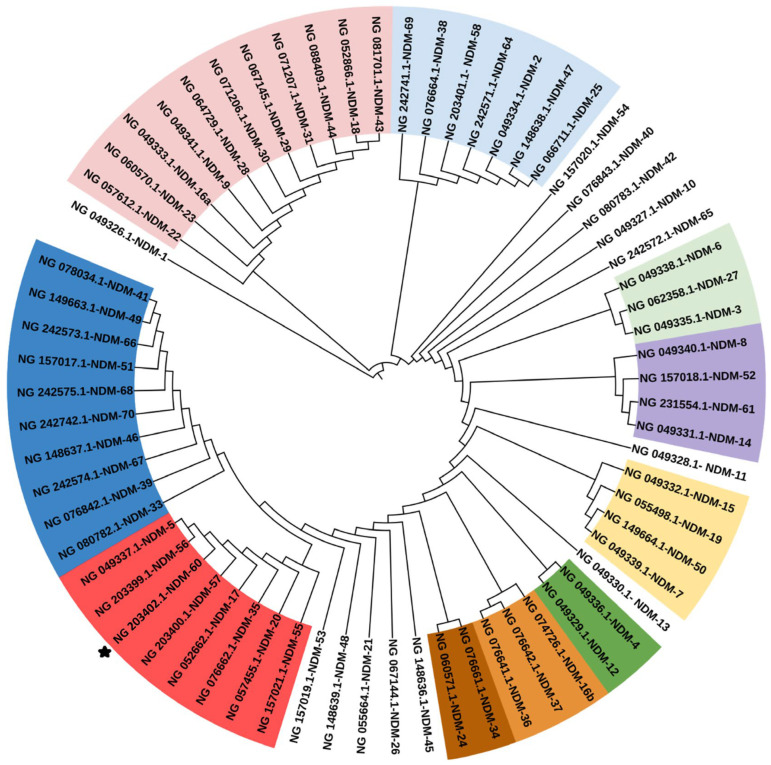
Phylogenetic analysis of *bla*_NDM_ alleles. The circular dendrogram depicts the evolutionary relationships among different NDM gene alleles, with each branch representing a distinct allele. NDM-60 is included in the red-colored cluster and marked with a star.

As the strain harbored a new NDM variant, we explored the phylogenetic relationship of this allele to all other known NDM alleles, totaling 67 nucleotide sequences, as shown in Figure 1. The tree is color-coded based on phylogenetic clusters, highlighting the genetic diversity and potential evolutionary relation within the *bla*_NDM_ gene family. The evolutionary history was inferred using the neighbor-joining method [18], and the evolutionary distances were computed using the maximum composite likelihood method [19]. As illustrated in the figure, the NDM-60 gene is closely related to both NDM-5 and NDM-56. NDM-5 differs from NDM-1 by two nucleotides at position 262 (G → T) and position 460 (A → C). Within the cluster containing NDM-5, most alleles vary by only a single nucleotide relative to NDM-5. Specifically, NDM-60 includes the two nucleotide differences seen in NDM-5, with an additional variation at position 604 (G → A). Similarly, other related alleles exhibit specific single-nucleotide variations, including NDM-56 at position 260 (T → G), NDM-55 at position 259 (G → A), NDM-57 at position 5 (A → G), NDM-17 at position 508 (G → A), NDM-35 at position 251 (G → A), and NDM-20 at position 809 (G → A).

**Figure 2:**
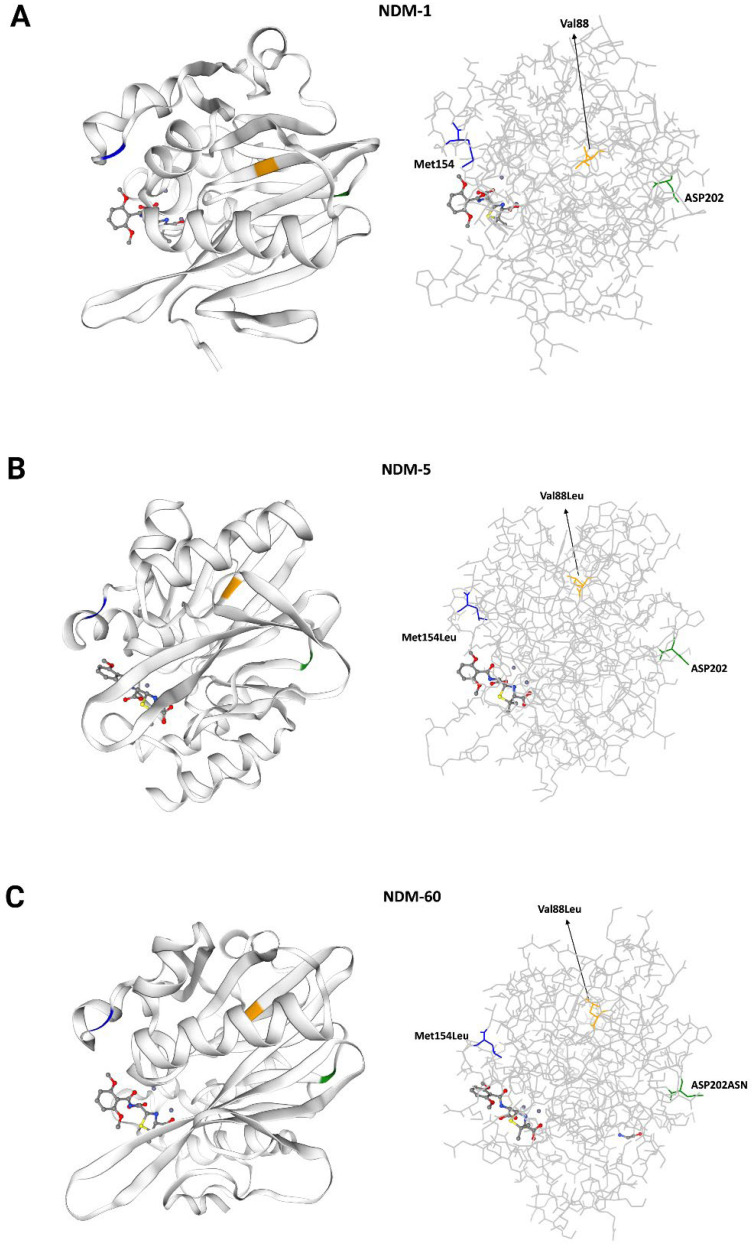
Protein homology model showing the differences between NDM variants: NDM-1 (**A**), NDM-5 (**B**), and NDM-60 (**C**). The protein backbone is shown as a ribbon/line. The three amino acid substitutions (Val88Leu in yellow, Met154Leu in blue, and Asp202Asn in green) in NDM-60 compared to NDM-1 are shown and labeled (**C** vs. **A**). Val88Leu and Met154Leu were also found in NDM-5 compared to NDM-1 (**B** vs. **A**).

At the protein level, NDM-60 contains an Asp202Asn substitution (Figure 2) compared to NDM-5. Additionally, two other amino acid substitutions, Val88Leu and Met154Leu, were identified in NDM-60 when compared to NDM-1, both of which are also present in NDM-5.

### 2.3. Plasmid Analysis and NDM Gene Localization

**Figure 3:**
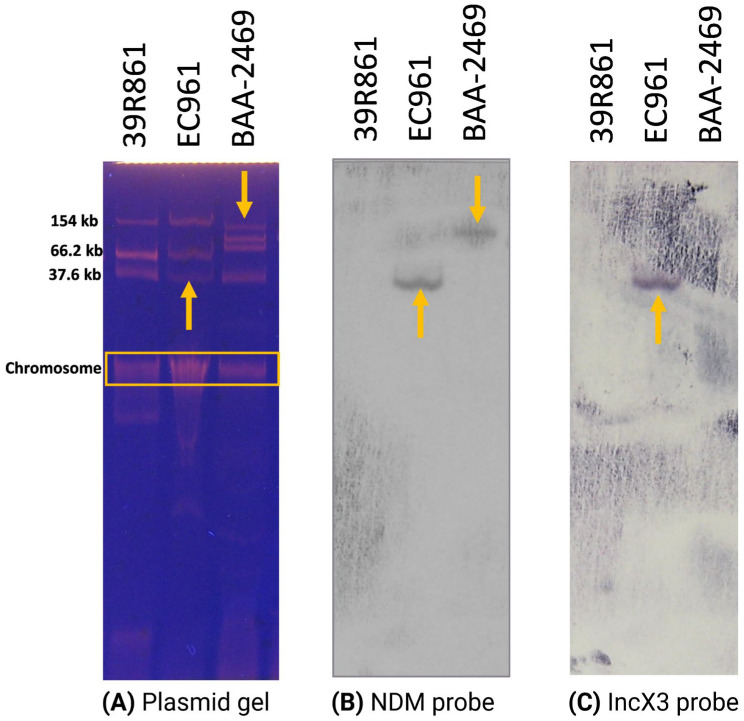
Gel electrophoresis and Southern blot analysis of plasmid profiles from *E. coli* strains EC961 (harboring NDM-60) and BAA-2469 (harboring NDM-1). Plasmid gel image (**A**) shows the results of agarose gel electrophoresis, displaying plasmid profiles for each isolate, with band sizes indicated. *E. coli* strain 39R861 was used as a size marker control (**A**). (**B**,**C**) Southern blots of the same gel, hybridized with a probe specific for NDM gene (**B**), then hybridized with a probe specific to the IncX3 plasmid replicon (**C**) to localize the IncX3-bearing plasmid. Yellow arrows highlight bands where successful hybridization occurred, confirming the presence of NDM-carrying plasmids at anticipated sizes in the analyzed strains and confirming that the same plasmid in EC961 carried the IncX3 plasmid replicon. BAA-2469 is lacking IncX3 based on ATCC records; thus, it was used as a negative control in plasmid replicon experiment (**C**), while it worked as a positive control for the NDM gene (**B**).

WGS showed that EC961 carried three plasmid types, including IncFIA, IncI (Gamma), and IncX3, which was also revealed by plasmid analysis on agarose gel showing three plasmid bands, with approximate sizes of 150, 66, and 36 kb (Figure 3). We tried to identify the location of the NDM-60 gene and if it is transmissible by conjugation. Southern blotting and hybridization analyses confirmed that the NDM-60 gene is harbored on an IncX3 plasmid of approximately 36 kb (Figure 3). Attempts to transfer this plasmid via conjugation to recipient strains were unsuccessful across multiple assays, indicating limited conjugative transfer potential under the conditions tested.

### 2.4. Comparative Genomic Analysis with the Single Reported Strain with NDM-60 and Closely Related NDM Types

**Figure 4:**
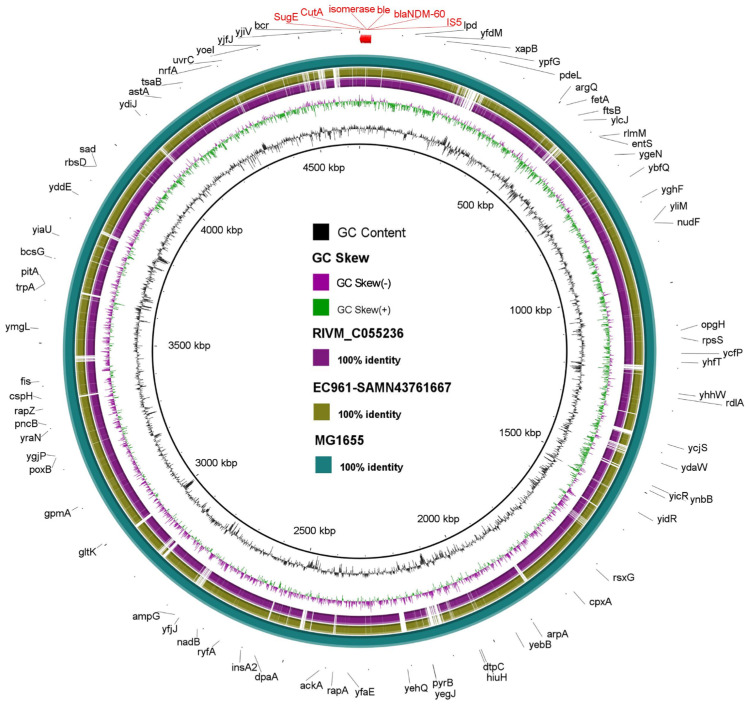
Comparative genomic analysis of *Escherichia coli* strain EC961 (SAMN43761667) against strain RIVM_C055236 (SAMN39950546) (carrying NDM-60 gene) and MG1655 *E. coli* reference strain (NC_000913.3). The innermost ring (black) displays the GC content, while the next ring (green and purple) represents the GC skew (green for positive skew, and purple for negative skew). The outer colored rings depict the sequence identity compared to the reference genomes, with colors indicating 100% identity. The NDM-60 gene, associated with carbapenem resistance, is highlighted in red on the outermost ring, along with key annotated genes. These labeled genes are positioned according to their location on the genome, facilitating the visualization of genomic similarities and differences between the strains. Black-colored genes represent antibiotic resistance and survival traits.

We performed comparative genomic analysis of all known NDM-60-containing strains. Notably, the NDM-60 gene was reported once by a research team from the National Institute for Public Health and the Environment in the Netherlands in July 2023. The gene was identified in *Escherichia coli* strain RIVM_C055236, which was found to have the subclass B1 metallo-beta-lactamase NDM-60 gene according to the NCBI database (GenBank accession number: OR139852.1) [20]. The whole-genome sequence of this strain was retrieved from the EnteroBase database (https://enterobase.warwick.ac.uk/species/index/ecoli, accessed on 2 August 2024), which is also available from the NCBI (BioSample: SAMN39950546; SRA: SRS20469100; Bio Project: PRJNA1076808). According to the NCBI, the specimen from which the strain was isolated was a swab from 66-year-old male patient hospitalized in the Netherlands [21]. Figure 4 shows the comparative genomics analysis of our strain (EC961) and RIVM_C055236, highlighting the high genetic similarity of the strains.

This circular genome map provides an in-depth comparative analysis of the EC961 strain, highlighting its genetic features, metabolic adaptations, and resistance mechanisms that collectively contribute to its resilience and potential pathogenicity. The map compares the EC961 strain to reference genomes RIVM_C055236 and MG1655, illustrating regions of genetic similarity and divergence, with a particular emphasis on antibiotic resistance and survival traits. Beyond its resistance elements, the strain’s metabolic machinery is robustly equipped for efficient energy utilization and stress adaptation, as shown by the genes highlighted in black.

**Figure 5:**
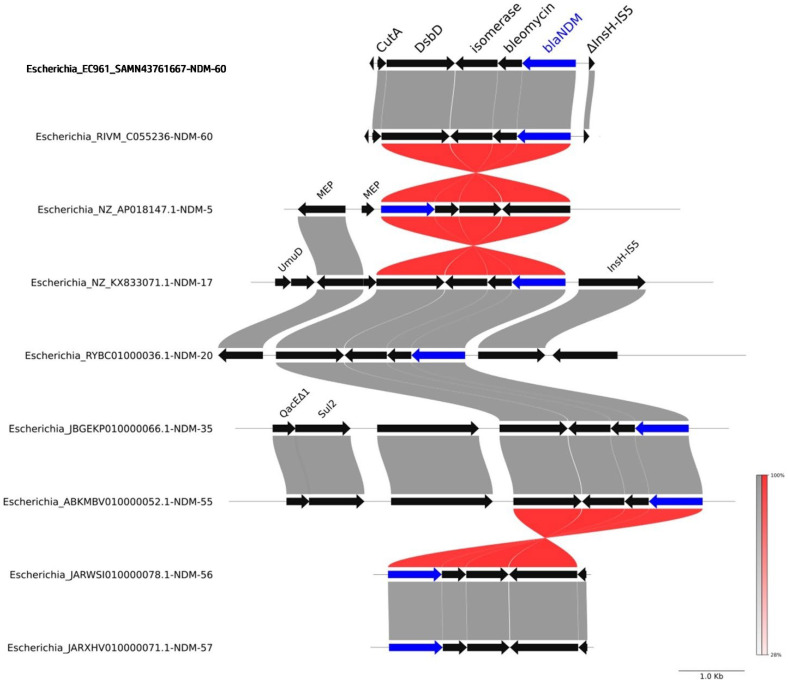
Comparative genomic analysis of NDM gene context across various *Escherichia coli* isolates. The figure displays the genetic environment surrounding different NDM alleles, including NDM-5, NDM-17, NDM-20, NDM-35, NDM-55, NDM-56, NDM-57, and NDM-60. Key genes associated with the NDM gene are highlighted. The red and gray shadings indicate regions of sequence homology and potential gene rearrangements, respectively. Abbreviations: CutA: periplasmic divalent cation tolerance protein; DsbD: cytochrome c-type biogenesis protein DsbD, protein-disulfide reductase; Isomerase: Phosphoribosylanthranilate isomerase; Bleomycin: bleomycin-resistance protein; InsH-IS5: transposase InsH for insertion sequence element IS5; MEP: mobile element protein; QacEΔ1: small multidrug resistance (SMR) efflux transporter; QacEΔ1, quaternary ammonium compounds; UmuD: error-prone repair protein; Sul2: dihydropteroate synthase type-2-sulfonamide resistance protein.

Furthermore, we compared the genetic context of NDM-60 in both strains with other related NDM alleles (shown in Figure 1), including NDM-5, NDM-56, NDM-17, NDM-55, NDM-57, NDM-20, and NDM-35. As shown in Figure 5, the genetic environment of NDM-60 is the same for both strains EC961 and RIVM_C055236. The genes downstream NDM are similar for the related alleles, but the difference is in the genes upstream to NDM. For NDM-60, there is a truncated InsH-IS5, i.e., transposase InsH for insertion sequence element IS5. There are regions of sequence homology and potential gene rearrangement events in the context of various NDM alleles.

### 2.5. Comparative Genomic Analysis with Strains Belonging to Sequence Type (ST) 940

**Figure 6:**
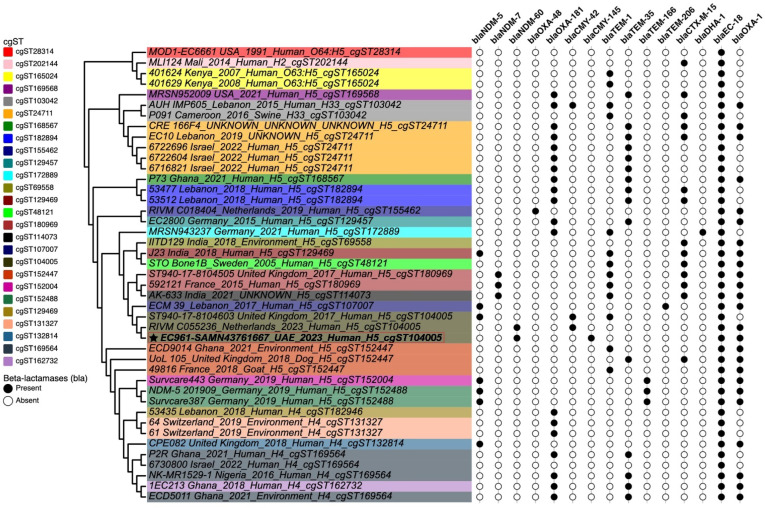
Core genome SNP-based phylogenetic tree of *E. coli* strains belonging to ST940 (*n* = 42) in comparison with strain EC961 from the same ST. The tree highlights evolutionary relationships based on SNPs and is color-coded according to cgST type. Detailed annotations include strain name, geographic origin, year of isolation, source (human/animal/environment), OH serotype, and cgST type. The presence of beta-lactamase genes, including CMY, TEM, CTX-M, DHA, EC, OXA-1, and carbapenemases (NDM, and OXA-48-like), is indicated with circles: black representing presence and white indicating absence. Our strain EC961 from the UAE is marked with a black star.

Genomic analysis indicated that our strain (EC961) belongs to sequence type (ST) 940; therefore, we retrieved the whole-genomic sequences of *E. coli* with the same ST to explore any genetic similarities among clonally related strains. The process involved screening 39,034 *E. coli* genomes from the GenBank database (https://ncbi.nlm.nih.gov/datasets/genome/?taxon=1, accessed on 20 August 2024). Finally, ST940 was detected in 42 strains, including the strain from the Netherlands (RIVM_C055236). Among the ST940 genomes, 26 have cgMLST profiles, with cgST24711 being the most prevalent (*n* = 5). Figure 6 shows the core genome SNP-based phylogenetic tree of all 43 *E. coli* strains belonging to ST940, with their geographic origin, year of isolation, source (human/environment), OH serotype, cgST type, and beta-lactamase genes.

Geographically, ST940 isolates are distributed across multiple continents, with 2 from North America, 10 from Africa, 14 from Asia (including EC961), 16 from Europe, and 1 from an unknown location. Lebanon has the highest number of ST940 isolates (*n* = 6), followed by Germany and Ghana (*n* = 5 each). Most of these strains are human isolates, with some environmental and a few animal isolates.

Notably, the ST940 strains exhibit significant diversity in their beta-lactamase gene content, with OXA-181 detected as a key resistance gene, while fewer isolates with NDM-5, NDM-7, NDM-60, OXA-48, and CTX-M-15 appear across multiple isolates. The detection of multiple TEM variants (TEM-1, TEM-35, TEM-166, and TEM-206) further emphasizes the broad spectrum of resistance mechanisms in these strains, particularly against beta-lactam antibiotics, including carbapenems and third-generation cephalosporins. All ST940 isolates carry the EC-18 gene, demonstrating a consistent resistance mechanism across this lineage. Certain resistance genes are unique to specific isolates, like OXA-48, which has only been detected in a human strain from the Netherlands, isolated in 2019 (RIVM_C018408); DHA-1 was found exclusively in a human isolate from Germany in 2021; TEM-206 was identified in a human isolate from Lebanon in 2017. Strain EC961 shares the same core genome sequence type (cgST) with other isolates from the Netherlands and the U.K. Notably, the Netherlands isolate, which also harbors the *bla*_NDM-60_ gene, carries an additional resistance gene, *bla*_CMY-42_, while *bla*_CMY-145_ is uniquely present in the UAE isolate EC961.

### 2.6. Resistome and Virulome of Strain EC961 Harboring NDM-60

In addition to beta-lactamase genes (NDM-60, CMY-145, OXA-1 and EC-18), EC961 harbors multiple resistance genes such as *mph(A)*, and *mdf(A)* for macrolide resistance, *erm(B)* for lincosamide, *tet(B)* for tetracycline, *dfrA* for trimethoprim, *catA1* for phenicol, *aadA1* for aminoglycosides, *sat2* for streptothricin, and *ble* for bleomycin. Additionally, it has several mutations responsible for lowering susceptibility to fosfomycin (*glpT*: E448K), quinolones (*gyrA*: D87N and S83L, *parC*: S80I, and *parE*: S458A), in addition to an insertion mutation in penicillin-binding protein 3 (PBP3 *ftsI* gene: N337NYRIN). The strain also has a mutation in *pmrB* (Y358N), which is involved in modifying the bacterial outer membrane by adding positively charged groups to lipopolysaccharide, thus reducing colistin binding.

Additionally, it has multiple virulence factors across various functional categories (Table 2). Within the adherence category, genes associated with CFA/I fimbriae are present. Additionally, *csg* genes were detected, which encode curli nucleator protein, which triggers the polymerization of Csg to form curli fibers on the cell surface, facilitating biofilm formation. Additionally, genes related to *E. coli* laminin-binding fimbriae (ELF) were detected. In contrast, genes associated with other fimbrial structures, such as P fimbriae, S fimbriae, and Type I fimbriae, are absent. In the invasion category, positive results were observed for *ibeB* and *ibeC*, genes associated with the invasion of endothelial cells. Genes involved in iron uptake, including siderophores; enterobactin/ferric enterobactin esterase gene (*fes*) and ferrienterobactin transporter genes were also detected. Secretion system analysis indicated the presence of several type VI secretion system (T6SS) genes, specifically within the ACE and SCI-I clusters. Type III secretion system effectors were also found, in addition to genes encoding the general secretion pathway proteins. The toxin profile was limited, with only hemolysin/cytolysin A (*hlyE/clyA*) detected, while other toxins, such as cytolethal distending toxin and heat-labile enterotoxin, are absent. For anti-phagocytosis and serum resistance, capsule biosynthesis gene (*wzi*) is present, while other antiphagocytosis genes were not detected. In the lipid and fatty acid metabolism category, a positive result was observed for the pantothenate synthesis (*panD*) gene.

## 3. Discussion

Recent reports highlight the emergence of evolving NDM variants with enhanced carbapenemase activity [22]. The rapid evolution of these variants complicates the clinical management of infections due to high resistance rates, thereby compounding the threat to human health [23]. In this study, we report the emergence of a novel NDM variant (*bla*_NDM-60_) in the UAE. Globally, this variant was reported once only in the Netherlands, with a lack of published literature describing this NDM allele and its genomic context. Thus, this report is the first of its kind deciphering this new NDM type and elucidating its genetic context, with full genomic characterization of the harboring strain.

The strain described in this work exhibited high resistance levels to multiple antibiotics including novel drugs, such as the innovative beta-lactamase inhibitors coupled with potent beta-lactams. This is indeed alarming due to the extensive resistant nature of the strain, which can be attributed to the potent hydrolytic effect of NDM-60, hence its resistance to inhibition by the most potent beta-lactamase inhibitors. It is worth noting that the four-amino-acid (YRIN) insertion mutation in PBP3, the major target of beta-lactams, was found in our strain. This mutation is associated with low susceptibility to cephalosporins and monobactams [24] and is known for its ability is to cause resistance even to novel combinations with new beta-lactamase inhibitors, especially in *E. coli* strains expressing the NDM gene [25,26]. Other beta-lactam resistance genes such as CMY-145, a potent AmpC beta-lactamase, were detected in this strain. Research on *E. coli* has shown that the overexpression of β-lactamases from class C, such as CMY, can contribute to carbapenem resistance, especially when coupled with other resistance mechanisms [27]. Among the global strains from ST940, our strain is the only one harboring CMY-145, indicating the uniqueness of this strain. Our strain is distinct from the closely related strain with NDM-60 from the Netherlands, which has CMY-42. These enzymes are becoming more prevalent in *E. coli* strains in multiple parts of the world [28] but are still new to the Middle East [29].

Variable responses to the novel combinations of β-lactam/β-lactamases inhibitors were noticed in our strain. Diazabicyclooctanes (DBOs), such as nacubactam and zidebactam, are novel β-lactam-like molecules [30] that are still in clinical development [31]. They have three possible mechanisms of action by acting as β-lactamase inhibitors, as PBP2-targeting agents, and as enhancers of the activity of several β-lactams targeting PBP3 [32]. For meropenem/nacubactam and cefepime/zidebactam, the strain is susceptible to the former but resistant to the latter. This can be partially attributed to the nature of DBOs in the combinations, as zidebactam is superior to nacubactam as a β-lactamase-independent enhancer targeting PBP2 [33,34,35]. It is also possible that the coupled β-lactam (meropenem vs. cefepime) affected the success of the synergistic combination in inhibiting bacterial growth, especially with the expression of NDM-60, which can degrade β-lactam drugs and resist inhibition by the novel DBOs.

Novel bicyclic boronates, such as taniborbactam and xeruborbactam, are the most recent compounds currently undergoing clinical trials for use in combating MBL-producing CREs. They are both considered pan-spectrum BLIs due to their ability to inhibit all β-lactamases from Classes A to D, including MBLs [36,37]. We noticed that our NDM-60-carrying strain is resistant to the combination with taniborbactam (cefepime/taniborbactam) but not to the one with xeruborbactam (meropenem/xeruborbactam). Overall, xeruborbactam was successful in inhibiting all NDM variants based on recent research, which is not true for taniborbactam, as some NDM types showed reduced susceptibility [24]. A recent report shows that NDM-9 escapes the inhibitory action of taniborbactam by a single amino acid substitution (Glu149Lys) as compared to NDM-1 [38]. Docking simulation studies showed that the Lys residue has a disruptive role in the interaction between NDM-9 and taniborbactam. This phenomenon is not exclusive for NDM-9 but can be seen in other NDM variants (such as NDM-30) if the location of the amino acid substitution(s) affects the negatively charged NDM side chain interacting with antibiotics. These changes alter the conformation and electrostatics of the active site of NDM [39].

Structurally, NDM-60 was found to be closely related to NDM-5, with only an extra amino acid substitution (Asp202Asn),l but both variants have two amino acid substitutions (Val88Leu and Met154Leu) compared to NDM-1. These substitutions may contribute to NDM-60’s increased carbapenemase activity. It has been shown that NDM-5 and its closely related variants with additional substitutions, such as NDM-17 (Glu170Lys) and NDM-20 (Arg270His), exhibit stronger carbapenemase activity compared to NDM-1 [40]. Similarly, the MICs of the carbapenems for NDM-5-producing isolates were reported to be higher than those of NDM-1 producers [41]. The Val88Leu substitution was reported to decrease the hydrolytic activity of NDM, while the Met154Leu substitution appeared to increase the carbapenemase activity [40,42]. This is in line with our findings, as our NDM-60-carrying strain exhibited higher MICs than the control strain with NDM-1 in terms of reduced susceptibility to beta-lactams and combinations with beta-lactamases. Distinct amino acid substitutions identified in some NDM variants are located outside the active site [43]; nevertheless, these variants have been reported to exhibit altered activities against β-lactams [15]. The unique amino acid substitution in NDM-60 (Asp202Asn) is located outside the recognized active sites for NDM. Further research is required to explore if it has a significant impact on enzyme activity.

By analyzing the bacterial genomes of our strain, we discovered a wide array of genes linked to resistance to multiple antibiotics. Additionally, it has a repertoire of virulence genes helping the bacteria in the colonization of and establishment in the host. The virulence factors responsible for adherence and invasion, iron acquisition, and toxins enable bacteria to survive for longer periods in colonized hosts, thereby increasing their chances of eventually causing infection [44]. Furthermore, the presence of biofilm-associated genes helps the bacteria form biofilms, which can prolong bacterial survival, contribute to long-term colonization, and increase antimicrobial resistance [45]. This sheds light on the complexity of AMR problem, as new variants of NDM are emerging in highly resistant and virulent strains, further complicating the management of infections caused by these bacteria [23].

Remarkably, molecular typing revealed a great diversity of STs among NDM-positive strains, with some clones classified as healthcare-associated or international high-risk lineages, including *E. coli* ST10, ST131, ST167, and ST410 [13]. These high-risk clones have gained medical notoriety due to their successful spread and persistence in human healthcare settings worldwide, being frequently responsible for life-threatening infections and presenting the genetic convergence of virulence and AMR genes. In our analysis investigating a huge collection of *E. coli* genomes from all over the world, we found a few strains (*n* = 42) worldwide belonging to ST940, in addition to ours. This reflects the limited global dissemination of this clone. Nevertheless, the isolates were recovered from diverse geographical locations, suggesting sporadic distribution. Furthermore, cgST types are highly variable among these strains, suggesting high diversity. Notably, ST940 strains exhibit significant diversity in their beta-lactamase gene content, suggesting continuous evolution of the clone in different parts of the world with additional resistance capabilities that make treatment even more challenging. Intriguingly, not all ST940 strains carry an NDM gene, and those which are positive carry either NDM-5 or NDM-7, except the strain from the Netherlands, which carries NDM-60. The rest of the strains have mostly OXA-48-like genes, which are more common than NDM among the strains. The majority of these strains are human isolates, though the presence of environmental and animal isolates suggests additional reservoirs for these genes contributing to the persistence and spread of these resistant clones [46,47].

NDM spread is not usually associated with dominating clonal strains; rather, it is mediated by several different plasmid incompatibility (Inc) types [48]. It has been reported that the *bla*_NDM_ gene is carried by various types of plasmids such as IncC, IncB/O/K/Z, IncFIA, IncFIB, IncFIC, IncFII, IncFIII, IncHI1, IncHI2, IncHI3, IncN, IncN2, IncL/M, IncP, IncR, IncT, IncX1, IncX3, IncX4, IncY, ColE10, and IncA/C [15,23]. It is rarely found to be chromosomally integrated [49]. Multiple variants such as NDM-1, NDM-5, NDM-7, NDM-9, NDM-17, and NDM-20 have been detected on pandemic plasmids, such as IncX3 and IncF-type [13,15], with more reports pointing to the dominance of IncX3 plasmids as NDM carriers [15]. These findings suggest this plasmid’s important role as a vehicle of gene transport and evolution. In our study, NDM-60 was also found to be located on IncX3 plasmid, as confirmed by Southern blotting analysis. The plasmid was not conjugative, despite our repeated attempts. It is possible that genome reassortment and recombination events affected the conjugative machinery of this plasmid [50]. This is supported by the results of examining the genetic context of *bla*_NDM_. A truncated transposase InsH for insertion sequence element IS5 was found upstream of *bla*_NDM-60_. This interruption can be attributed to the insertion of many other mobile genetic elements such as IS1, IS5, and IS26 with subsequent recombination [51]. Previous reports showed the presence of a disrupted insertion sequence ISAba125 (ΔISAba125) by the insertion of an IS5 element, which results in a truncated transposase [52]. A similar genetic environment has been observed in many NDM-positive isolates, with two common features, namely, the presence of the insertion sequence ISAba125 (intact or truncated) upstream of *bla*_NDM_, and the downstream regions of the NDM gene mostly have a bleomycin resistance gene [48] and genes encoding a putative phosphoribosylanthranilate isomerase and the oxidoreductase DsbC superfamily protein [15].

Previous studies have shown that most strains carrying the *bla*_NDM_ gene were isolated from patients attending intensive care units (ICUs), who usually have longer hospital stays, which increases the risk of infections and the evolution of CRE pathogens [48]. In this study, the strain was recovered from a rectal swab of a patient screened for CRE carriage during hospitalization. Interestingly, the other NDM-6-carrying strain from the Netherlands was also isolated from swab [21], and the first strain carrying the most closely related NDM type (NDM-5) was isolated for the first time in the U.K. from routine screening swabs of perineum and throat [41], suggesting possible colonization with strains harboring these NDM variants.

Gut colonization with MDR *Enterobacterales* has been reported before [53]. Asymptomatic intestinal colonization could also favor the transmission of AMR genes to other subjects and can predispose the carriers to serious endogenous infections. The gut microbiota is a resident rich and dynamic reservoir that has been shown to be the major source of MDR bacteria in hospitalized patients. The rates of colonization with ESBL and CRE bacteria ranged between 12 and 65% in different countries among the population of hospitalized patients [53]. This is true in the community as well, as colonization with MDR *Enterobacterales* among healthy subjects has reached high levels, showing a clear disparity between high-income and low-income countries, with significantly higher colonization rates in developing nations.

Interestingly, interspecies plasmid transmission in the human gut has been shown in studies reporting co-colonization of different species harboring different carbapenemases [54]. Therefore, the human gut represents a natural incubator for microbes, favoring the evolution of MDR pathogens [55]. During their treatment, the frequent use of potent broad-spectrum antibiotics such as carbapenems, tigecycline, or piperacillin/tazobactam poses a high risk for CRE proliferation due to microbiota dysbiosis [56]. Under antibiotic stress, gut microbiota dysbiosis gives CREs a competitive advantage by eliminating susceptible members of the gut microbiota, which promotes CRE growth and colonization [57]. This underscores the demand for proper intestinal CRE screening and the possibility of employing new methods for AMR prevention by modulating the gut microbiome as it can be a source of AMR pathogens.

## 4. Materials and Methods

### 4.1. Bacterial Collection and Identification of the Strain Harboring NDM-60

In this study, we investigated a collection of clinical strains of *E. coli* (*n* = 75) isolated from patients attending Tawam hospital, Al-Ain in Abu Dhabi, UAE. Bacterial identity as *E. coli* was confirmed using a VITEK 2 system (BioMérieux, Craponne, France) [58]. We screened the strains using whole-genome sequencing (WGS). During the bioinformatic analyses, we identified a new NDM variant in one of the strains (coded as EC961). Subsequently, the clinical data of the patient infected with the strain were retrieved from the medical records.

### 4.2. Antibiotic Susceptibility Testing

Antibiotic susceptibility testing was conducted according to the latest Clinical Laboratory Standards Institute (CLSI) guidelines. As a quality control, we used *E. coli* strains ATCC25922 and BAA-2469 obtained from the American Type Culture Collection (Microbiologics, St. Cloud, MN, USA) [59]. Mueller–Hinton broth, obtained from Oxoid, Hampshire, UK, was used for the assessment of the minimum inhibitory concentration (MIC) with a broth microdilution test for selected antibiotics, including meropenem, ertapenem, imipenem, zidebactam, nacubactam, cefepime, colistin, tigecycline, ceftazidime, cefotaxime, aztreonam, ciprofloxacin, gentamicin, and amikacin. Furthermore, novel β-lactamase inhibitors combined with β-lactams were tested including ceftazidime/avibactam, cefepime/zidebactam, cefepime/taniborbactam, meropenem/xeruborbactam, and meropenem/nacubactam. The novel siderophore-cephalosporin (cefiderocol) was tested, and MIC was determined in iron-depleted cation adjusted BBL^™^ Mueller Hinton II Broth (Becton Dickinson, Franklin Lakes, NJ, USA), prepared as per the CLSI guidelines, as described in our previous work [60]. Tigecycline and colistin broth microdilution results were interpreted based on the European Committee on Antimicrobial Susceptibility Testing (EUCAST) guidelines [61], while the rest of the results were interpreted based on the latest CLSI guidelines [59].

Based on the MIC results, the strain was classified as CRE due to resistance to all tested carbapenems (meropenem, ertapenem, and imipenem) according to the cut-off points of MICs specified by the CLSI guidelines [59].

### 4.3. Whole-Genome Sequencing (WGS) and Bioinformatic Analyses

DNA was extracted using a Wizard^®^ Genomic DNA Purification Kit (Promega, Madison, WI, USA) as recommended by the manufacturer. Quality and quantity of DNA were explored with a nanodrop (Thermo scientific, Waltham, MA, USA). Fluorometric quantification was also performed with a Qubit 2 (Thermo Scientific, Waltham, MA, USA). WGS was performed using DNBSEQ-G400RS (DNA nanoball sequencing platform; MGI-Tech, Hong Kong) at a coverage of >200X. FASTQ files were checked for quality using the FastQC tool (http://www.bioinformatics.babraham.ac.uk/projects/fastqc/, accessed on 4 August 2024). De novo assembly was accomplished for the paired-end sequence reads using unicycler (https://github.com/rrwick/Unicycler, accessed on 4 August 2024), with further quality assessment using QUAST v5.0.2 (https://quast.sourceforge.net/quast, accessed on 4 August 2024) [62,63]. Sequences were annotated using the rapid prokaryotic genome annotation tool (Prokka) [64,65].

The assembled genome was examined for antibiotic resistance genes, sequence type, and plasmid replicon types. Antibiotic resistance genes were identified using the Comprehensive Antibiotic Resistance Database (CARD) database (https://card.mcmaster.ca/, accessed on 14 August 2024) [66]. Sequence type (ST) was determined using the genomic sequence to query the *E. coli* MLST database (http://enterobase.warwick.ac.uk/species/index/ecoli, accessed on 14 August 2024) [67].

Plasmid incompatibility types were obtained by a plasmid finder provided by the Center for Genomic Epidemiology (https://cge.food.dtu.dk/services/PlasmidFinder/, accessed on 14 August 2024). Virulence genes were detected using the Virulence Factor Database (VDFB), available at http://www.mgc.ac.cn/VFs/, accessed on 14 August 2024 [68].

### 4.4. Core Genome Analysis

As EC961 belongs to ST940, we aimed to elucidate the evolutionary relationships among the strains of *E. coli* belonging to the same sequence type (ST940). We downloaded all available complete or draft genome sequences of ST940 from NCBI/GenBank (41 genomes, as of 20 August 2024) and one additional genome from EnteroBase (https://enterobase.warwick.ac.uk/species/index/ecoli, accessed on 14 August 2024). Core genome single-nucleotide polymorphism (SNP) alignments were generated using Snippy v4.6 (https://github.com/tseemann/snippy, accessed on 20 August 2024), and recombination was filtered using Gubbins v2.4.1 [69]. A maximum likelihood phylogenetic tree was inferred using FastTree v2.1 [70], and the resulting tree was visualized, annotated, and edited using Evolview v3 (https://academic.oup.com/nar/article/47/W1/W270/5494715, accessed on 15 October 2024). To further analyze the presence of acquired antimicrobial resistance genes, the genome sequences of all ST940 strains (*n* = 43) were screened using ResFinder v4.6 (https://pubmed.ncbi.nlm.nih.gov/32780112/, accessed on 14 August 2024).

### 4.5. Conjugation Assay

We aimed to test the transmissibility of *bla*_NDM-60_; thus, a conjugation experiment was performed using *E. coli* J53RAZ (a Na-azide resistant derivative of the rifampin-resistant *E. coli* J53 K-12) as the recipient strain. Log-phase cultures of the donor (EC961) and recipient strains were combined in a 1:4 ratio in a sterile 50 mL Falcon tube, then fresh media were added to a final volume of 10 mL. The mixture was incubated without shaking overnight. The next day, bacterial growth was collected by centrifugation at 3500 rpm for 15 min. The resulting pellet was resuspended in 5 mL of 1X PBS, centrifuged, washed once with 1X PBS, and resuspended in 3 mL of PBS. This suspension was serially diluted and plated onto selective media containing 100 μg/mL Na-azide and either 8 mg/L ceftazidime or 2 mg/L meropenem for NDM selection. Plates were incubated overnight at 37 °C to observe any growth from transconjugants. Single colonies were then picked for further analysis and tested with PCR to verify the success of conjugation [71].

### 4.6. Plasmid Profiling and Gene Location Analysis by Southern Blotting

For routine plasmid detection and isolation, a modified Kado and Liu method was applied [72]. Bacterial cells were collected from approximately 4 cm^2^ of overnight TSA culture at 37 °C and suspended in 250 µL of lysing solution (3% SDS, 50 mM Tris, pH 12.57). The suspension was mixed gently until uniform and incubated at 60 °C for 45 min, with occasional mixing. Following incubation, 250 µL of a 1:1 phenol–chloroform solution was added and emulsified via gentle shaking. The solution was centrifuged at 13,000 rpm for 15 min, and the upper aqueous layer was carefully transferred to new tubes. Samples were run on a 0.8% agarose gel at 140 volts for 3 h, stained with ethidium bromide, destained with sterile Milli-Q water, and scanned using a Biometra gel documentation system (Analytik Jena, Jena, Germany).

Gene location analysis of NDM was performed by Southern blotting on plasmid gels prepared as described above and following the methods reported before [41]. The gels were sequentially depurinated in 0.25 M HCl, denatured in 0.5 M NaOH and 1 M NaCl, and neutralized in 1 M Tris and 0.6 M NaCl, each step lasting 15 min at room temperature with gentle shaking and interspersed with sterile water rinses. Gels were then capillary-transferred to Hybond N+ membranes (Roche, Penzberg, Germany) overnight in 20X SSC buffer and cross-linked with UV at 70,000 µJ. Hybridization probes were prepared by amplifying target genes (*bla*_NDM_, and IncX3) via PCR, followed by DNA purification and quantification. The purified DNA fragments were labeled using a DIG DNA labeling kit (Roche, Germany). Membranes were prehybridized at an optimal temperature calculated based on probe characteristics, then hybridized with the probe-containing buffer. After hybridization, membranes underwent a series of washes. Following hybridization, membranes were rinsed, blocked, incubated in an antibody solution, and treated with substrate in the dark until the desired signal intensity was achieved. Finally, blots were digitized using a Biometra system (Analytik Jena, Germany). For reprobing, membranes were stripped with dimethylformamide at 56 °C, then reprocessed from the prehybridization step. IncX3 probe was used. The BAA-2469 strain, obtained from the American Type Culture Collection (ATCC, Manassas, VA, USA), was used as a negative control because it is lacking the IncX3 plasmid replicon based on ATCC records (https://genomes.atcc.org/genomes/0505fb1208a84582?tab=annotations-tab, accessed on 2 October 2024). *E. coli* strain 39R861 was used as a plasmid size standard [73].

### 4.7. Phylogenetic Analysis of bla_NDM_ Alleles and NDM Protein Homology Modeling

Full-length NDM gene sequences were obtained from the NCBI database (https://www.ncbi.nlm.nih.gov/pathogens/refgene/#blaNDM, accessed on 26 October 2024), covering the whole range of NDM alleles. The NDM sequences were aligned using Molecular Evolutionary Genetics Analysis across computing platform (Mega X) [74] to ensure an accurate comparison of the nucleotide variations among the alleles. NDM alleles were also compared at the protein level to check for amino acid substitutions in NDM-60 relative to the other NDM types [15].

A phylogenetic tree was constructed using MEGA X using the neighbor-joining method [18]. The evolutionary distances were computed using the maximum composite likelihood method [19] and are reported in the units of the number of base substitutions per site. This analysis involved 67 nucleotide sequences. Codon positions included were 1st + 2nd + 3rd + noncoding. All ambiguous positions were removed for each sequence pair (pairwise deletion option). There was a total of 828 positions in the final dataset. The resulting phylogenetic tree was visualized in a circular dendrogram format using iTOL [75]. The branches were color-coded to represent different phylogenetic clusters, highlighting the evolutionary divergence of the *bla*_NDM_ alleles.

A protein model of NDM-60, compared to the closely related NDM-5 and prototypic NDM-1 proteins, was generated using the SWISS-MODEL platform, an automated ExPASy homology modeling server available at https://swissmodel.expasy.org/, accessed on 1 November 2024 [76].

### 4.8. Genetic Context Analysis of bla_NDM_ Alleles

Sequences containing *bla*_NDM_ alleles were retrieved from publicly available genomic databases (NCBI). The selected sequences included *bla*_NDM_ alleles from *E. coli* strains. The sequences were aligned and visualized using pyGenomeViz (https://github.com/moshi4/pyGenomeViz, accessed on 20 October 2024). *bla*_NDM_ genes and adjacent genes, such as *ble* (bleomycin resistance gene), isomerase, and reductase, were annotated. Red and gray shading was applied to indicate regions of homology [20].

## 5. Conclusions

We report the emergence of a new variant of NDM (*bla*_NDM-60_) for the first time in the UAE and in the Middle East. Given the high risk of MBL-mediated infections, the continuous evolution of NDM represents a significant concern at the global level. This is attributed to its enhanced carbapenemase activity and resistance to novel antibiotics, even combinations with potent beta-lactamase inhibitors. This is alarming due to the difficulty in treating these infections with limited therapeutic options. The presence of additional resistance mechanisms further complicates the problem of AMR in such strains. The molecular epidemiology investigation revealed that the strain belongs to ST940, which is sporadic but global in its distribution, with high diversity among the strains from the same ST, reflecting the ongoing evolution and adaptation of the MDR strains. Furthermore, NDM-60 is located on nonconjugative plasmid, which may be a factor limiting the distribution of this gene and the lack of reports on this variant. The isolation of NDM-60-carrying strains from screening swabs, particularly from the gastrointestinal tract, highlights the importance of the gut microbiota as a reservoir for resistant organisms and sheds light on the importance of discovering innovative approaches to avoid colonization with MDR pathogens and stop transmission in the community and healthcare settings. This also emphasizes the critical need for robust surveillance and genetic screening programs to detect novel variants. Indeed, there is an urgent need for the discovery and development of novel antibiotics to combat the emerging NDM variants. This requires extensive structural and functional studies of NDM variants to facilitate the design of effective novel inhibitors.

## Figures and Tables

**Table 1:** Minimum inhibitory concentrations (MICs; µg/mL) of various antibiotics for strain EC961 carrying NDM-60 compared to two control strains: BAA-2469, carrying NDM-1, and ATCC 25922, which is susceptible to all antibiotics. Red color: resistant; green color: susceptible; uncolored cells represent drugs with unknown susceptibility cut-off points based on Clinical and Laboratory Standards Institute (CLSI) and European Committee for Antimicrobial Susceptibility Testing (EUCAST) guidelines.

Antibiotics	Strains and Their MICs (µg/mL)		
	EC961	BAA-2469	ATCC 25922
Meropenem	>32	16	≤0.0625
Ertapenem	>32	32	≤0.0625
Imipenem	16	32	≤0.0625
Cefiderocol	4	1	0.5
Ceftazidime	>128	>128	≤0.25
Ceftazidime/Avibactam	>16	>16	≤0.25
Cefotaxime	>128	>128	≤0.25
Cefepime	>128	128	0.5
Aztreonam	128	32	0.5
Ciprofloxacin	>64	>64	≤0.125
Gentamicin	2	>128	1
Amikacin	2	>128	2
Tigecycline	0.125	0.25	≤0.0625
Colistin	<0.25	0.5	0.25
Zidebactam ^#^	0.5	0.25	0.125
Cefepime/Zidebactam	0.5	0.25	0.125
Nacubactam ^#^	>4	2	2
Meropenem/Nacubactam	16	2	≤0.0315
Cefepime/Taniborbactam	>16	1	≤0.0315
Meropenem/Xeruborbactam	1	0.125	≤0.0315

# Diazabicyclooctanes, such as nacubactam and zidebactam, were tested alone to check for their intrinsic antibacterial activity.

**Table 2:** Virulence gene distribution in *E. coli* EC961 isolate. Green color: gene present; red color: gene absent.

Virulence Factor Class	Related Genes	Presence	Absence
Adhesion			
CFA/I fimbriae	*cfaA*, *cfaB*, *cfaC*, *cfaD/cfaE*		
Curli fibers	*cgsD*, *cgsE*, *cgsF*, *cgsG*, *cgsA*, *cgsB*, *cgsC*		
*E. coli* laminin-binding fimbriae (ELF)	*elfA*, *elfC*, *elfD*, *elfG*		
P fimbriae	*papA*, *papB*, *papC*, *papD*, *papE*, *papF*, *papG*, *papH*, *papI*, *papJ*, *papK*, *papX*		
S fimbriae	*sfaA*, *sfaB*, *sfaC*, *sfaD*, *sfaE*, *sfaF*, *sfaG*, *sfaH*, *sfaS*		
Type I fimbriae	*fimA*, *fimB*, *fimC*, *fimD*, *fimE*, *fimF*, *fimG*, *fimH*, *fimI*		
Invasion			
Invasion of brain endothelial cells (Ibes)	*ibeB, ibeC*		
Iron Uptake			
Siderophores	*entB*, *entC*, *entD*, *entE*, *entE*, *entF*, *entS*		
Ferrienterobactin transporter	*fepA*, *fepB*, *fepC*, *fepD*, *febG*		
Enterobactin esterase	*fes*		
Secretion System			
ACE T6SS	*aec11*, *aec14*, *aec15*, *aec16*, *aec17*, *aec18*, *aec19*, *aec22*, *aec23*, *aec24*, *aec25*, *aec26*, *aec27/clpV*, *aec28*, *aec29*, *aec30*, *aec31*, *aec32*, *aec7*, *aec8*		
Type III secretion system effectors	*espL1*, *espR1*, *espX1*, *espX4*, *espX5*		
General secretion pathway	*gspC*, *gspD*, *gspE*, *gspF*, *gspG*, *gspH*, *gspI*, *gspJ*, *gspK*, *gspL*, *gspM*		
Toxins			
Cytolethal distending toxin	*cdtA*, *cdtB*, *cdtC*		
Heat-labile enterotoxin	*eltA*, *eltB*		
Hemolysin/cytolysin A	*hlyE/clyA*		
Antiphagocytosis			
Capsule biosynthesis	*wzi*		
Lipid and fatty acid metabolism			
Pantothenate synthesis	*panD*		

## Data Availability

Data were deposited in the Sequence Read Archive (NCBI) under Bio Project accession number PRJNA1160853 (SRR30660431; BioSample: SAMN43761667).
